# Optogenetic Gene Therapy for the Degenerate Retina: Recent Advances

**DOI:** 10.3389/fnins.2020.570909

**Published:** 2020-11-11

**Authors:** Michelle E. McClements, Federica Staurenghi, Robert E. MacLaren, Jasmina Cehajic-Kapetanovic

**Affiliations:** ^1^Nuffield Laboratory Ophthalmology, Department of Clinical Neurosciences, NIHR Oxford Biomedical Research Centre, University of Oxford, Oxford, United Kingdom; ^2^Oxford Eye Hospital, Oxford University Hospitals NHS Foundation Trust, Oxford, United Kingdom

**Keywords:** optogenetics, gene therapy, AAV, retinal degeneration, opsins

## Abstract

The degeneration of light-detecting rod and cone photoreceptors in the human retina leads to severe visual impairment and ultimately legal blindness in millions of people worldwide. Multiple therapeutic options at different stages of degeneration are being explored but the majority of ongoing clinical trials involve adeno-associated viral (AAV) vector-based gene supplementation strategies for select forms of inherited retinal disease. Over 300 genes are associated with inherited retinal degenerations and only a small proportion of these will be suitable for gene replacement therapy. However, while the origins of disease may vary, there are considerable similarities in the physiological changes that occur in the retina. When early therapeutic intervention is not possible and patients suffer loss of photoreceptor cells but maintain remaining layers of cells in the neural retina, there is an opportunity for a universal gene therapy approach that can be applied regardless of the genetic origin of disease. Optogenetic therapy offers such a strategy by aiming to restore vision though the provision of light-sensitive molecules to surviving cell types of the retina that enable light perception through the residual neurons. Here we review the recent progress in attempts to restore visual function to the degenerate retina using optogenetic therapy. We focus on multiple pre-clinical models used in optogenetic strategies, discuss their strengths and limitations, and highlight considerations including vector and transgene designs that have advanced the field into two ongoing clinical trials.

## Introduction

Optogenetics is a method that allows optical control of neural circuitry by ectopic expression of light-sensitive tools in target cells (Deisseroth et al., 2006). It offers a unique opportunity to treat inherited retinal degenerations of varied genetic origins with a universal therapeutic strategy. In a degenerate retina that has lost the light-sensitive photoreceptor cells, optogenetic therapy is a promising approach that combines neurobiology and genetic engineering techniques to provide light-mediated control over the cell physiology in surviving retinal cells that are normally insensitive to light. In a pioneering study, adeno-associated viral (AAV) vector-based delivery of a transgene encoding light-sensitive protein, channelrhodopsin (ChR2), was shown to be targeted to surviving cells of the retina, whereby its ectopic expression in cellular membranes converted the cells into artificial photoreceptors (Bi et al., 2006; Sahel and Roska, 2013). However, this landmark study highlighted several important challenges that needed to be addressed before optogenetics could be considered for human studies. The light levels needed for activation of the ChR2 sensor were very high and not encountered under normal light conditions. Thus, stimulation via external artificial light source would be necessary for this strategy to work as human therapy, but such high radiation levels are potentially toxic to the retina. In addition, being a microbial opsin, questions regarding safety and immune responses if used in humans needed to be answered. The strategy was also shown to have limited expression of the transgene in in the inner retina of a murine model of retinal degeneration and questions were raised about its translational potential when used in larger animal models and indeed human retina.

In this review we consider the multiple areas of research that have enabled development of optogenetic strategies for the treatment of inherited retinal degeneration, including vector design, transgene and opsin selection across various models of disease. Combined, the work reviewed highlight the great progress achieved in the field to date, which has led to two ongoing clinical trials with further upcoming human trials trials likely in the near future.

## Inherited Retinal Disease and the Degenerate Retina

**FIGURE 1:**
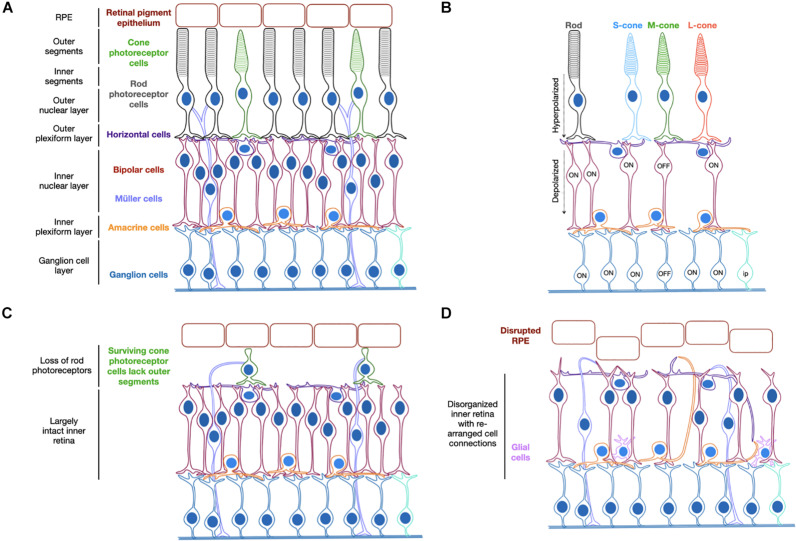
Schematic representation of the organized structure of a healthy retina **(A)**. Light activation of opsins triggers photoreceptor hyperpolarization, which causes depolarization of ON-bipolar cells with cone OFF-bipolar cells in the same receptive field hyperpolarized. The ON-ganglion cells are subsequently activated and the OFF-ganglion cells inhibited **(B)**. Degeneration begins by loss of rod photoreceptor cells and loss of outer segments on residual cone photoreceptor cells **(C)** followed by further cell loss and structural rearrangements in later stages of disease **(D)**. S/M/L-cone = cone photoreceptor cells containing short, medium or long wavelength-sensitive opsins. Ip = intrinsically photosensitive ganglion cell.

Inherited retinal degeneration can result from different mutations in more than 300 genes (RetNet: https://sph.uth.edu/retnet/) and is the leading cause of blindness in the working age population, affecting approximately 1 in 3,000 people world-wide (Sohocki et al., 2001). Clinical trials have shown encouraging success in recent years and have primarily focused on AAV vector gene supplementation strategies for particular forms of inherited retinal disease (Bainbridge et al., 2015; Russell et al., 2017; Xue et al., 2018; Cehajic-Kapetanovic et al., 2020b). However, such therapies are suitable for only a small subset of people suffering from inherited retinal disease. Many of the identified genetic causes have very low prevalence, which makes it unfeasible to develop gene-specific treatments for all forms and yet, regardless of the differences in genetic origin, the progression of disease occurs similarly in many patients. The process of retinal degeneration in the condition retinitis pigmentosa typically involves loss of rod photoreceptor cells followed by loss of cone photoreceptor cells with subsequent migration of retinal pigment epithelium into the inner retina (Figure 1). A healthy retina is highly structured and enables complex interactions between multiple cell types. When there is significant loss of photoreceptor cells, which form the outer retinal layer, cells of the inner retina continue to survive but over time can undergo remodeling. This refers to a change in the normal structure of the remaining retinal layers and includes synaptic rewiring, retraction of bipolar cell dendrites and changes to protein expression patterns and trafficking (Marc et al., 2003; Puthussery et al., 2009). Despite this, residual cells have been shown to maintain key molecular signatures and morphology, suggesting the surviving retina is a receptive environment for ectopic optogene expression (Jones et al., 2003).

The pattern of retinal degeneration is common across cases of retinitis pigmentosa regardless of the genetic origin and the strategy of optogenetic therapy therefore holds great potential as a universal treatment approach. It aims to target the surviving cell populations of the retina, which remain largely functional despite the structural changes that occur (Jones et al., 2016; Pfeiffer et al., 2020) and convert them to become light-sensitive through the provision of a light-sensitive opsin protein. The disease state of a given patient will determine the cell types that would most benefit from being targeted. For example, loss of rod photoreceptors occurs before cone photoreceptor cells, which can continue to survive for some time despite changes to their structure (Milam et al., 1998; Banin et al., 1999). In this scenario, encouraging light sensitivity in these residual cell types may prove beneficial. However, it may be the case that such cells are too few in number or too diseased to function following gene therapy and therefore the secondary layer of neurons, the bipolar cells, might prove a better target. In a healthy retina, the bipolar cells transfer the light signals received from photoreceptor cells to amacrine and retinal ganglion cells (Figure 1). Targeting these secondary neurons may therefore provide more opportunity for visual processing via the interconnected pathways than targeting the inner most cells of the retinal ganglion layer. Finally, in some instances of severe degeneration and retina remodeling, the retinal ganglion cells may be the desired cell targets. Hence there is justification for targeting different cell types of the degenerate retina for optogenetic therapy, which will be discussed further within this review.

## Relevant Models for Optogenetic Therapy

Developing optogenetic therapies requires pre-clinical testing in relevant *in vivo* and *ex vivo* models of inherited retinal disease (Table 1). The selection of model when investigating optogenetic therapies is critical to understanding their translational potential and may impact the degree of therapeutic efficacy observed. The efficacy of a particular optogenetic sensor may thus depend on the level of expression achieved in the target cell, which in turn can depend on the study model used. For example, studies with AAV-based reporter vectors indicate that cellular transduction profiles can vary depending on the mouse model used (Charbel Issa et al., 2013; van Wyk et al., 2017). Specifically, there are differences in transduction profiles achieved in healthy, wild-type retinas compared to disease models (Kolstad et al., 2010) or those with compromised barriers (Vacca et al., 2013). In addition, the stages of the disease in humans are mimicked in various animal models, both naturally occurring and transgenic in origin, and whilst preliminary data are often achieved in *rd1* mice for testing in eyes that reflect a late-stage degenerate retina, subsequent efficacy and long-term approaches may be better suited in a variety of models with different genetic origins and rates of disease. This would provide an indication on the potential and scope of optogenetic strategies, which are hoped to have universal application across patients with different causative mutations. The progression of retinal disease changes the global structure and function of the retina but at a cellular level, the genetic origin will impact the function of a given cell type. It may be that expanded testing across different mouse models, and indeed larger animal models, will confirm the promise of optogenetic strategies or provide evidence of preferred optogene selection for particular groups of patients. Current studies tend to be limited to testing in a single model when the scope of the field could be expanded by testing across multiple models. Different genes mean different mechanisms for disease and though the principle of optogenetics is to act as a universal approach, it needs to be confirmed that this is feasible.

**TABLE 1:** Summary of models that can be used in the pre-clinical assessment of optogenetic gene therapies.

**Model**	**Gene**	**Structural changes (P = postnatal day)**	**Functional changes**	**Strengths/Limitations**	**Model references**	**Optogenetic related studies**
**Murine models**						

**Naturally occurring**						
*Wild-type*	*e.g., C57BL/6*	Normal.	Normal.	Small ocular size with similar retinal structure and function compared to the primate retina but differing in size and inner limiting membrane thickness.	Shupe et al. (2006), Mohan et al. (2012)	Multiple studies (see main text).
*Rd1*	*Pde6b* Y347*	Rod photoreceptor degeneration begins at P8–P10 with complete loss by P21. Cone degeneration begins around P35 with continual loss up to P50, at which point a single row remains for up to 8 months of age.	Complete loss of scotopic ERG by P21 with loss of photopic ERG by P50.	Early onset, severe degeneration providing a model of late stage human retinal degeneration. Good for proof-of-principle assessments and for strategies aimed at end-stage disease. Offers a limited window of intervention for approaches that might be applied at earlier stages of disease and targeting residual cone photoreceptor cells.	Pittler et al. (1993), Farber et al. (1994), Hackam et al. (2004)	Multiple studies (see main text).
*Rd2/rds*	*Prph2* Large intron insertion leading to absence of protein	Photoreceptors lack outer segments at P7 and slow degeneration begins at P14 with complete loss of periphery at 9 months and central retina at 12 months.	ERG responses reduced but detectable with continual age-related decline and abolished by 12 months of age.	Slow, progressive rate of degeneration reflective of PRPH2 retinitis pigmentosa in humans, useful for optogenetic therapy safety and efficacy assessments in early stage retinal degeneration.	Reuter and Sanyal (1984), Jansen and Sanyal (1984), Travis and Hepler (1993), Ma et al. (1995)	Not to date.
*Rd6*	*Mfrp* Deletion of splice donor site and exon skipping	Subretinal deposits appear around P50. Photoreceptor degeneration occurs progressively with age with significant thinning at 7 months of age and complete loss at 24 months.	Abnormal rod and cone ERG responses are detected from P28 and show slow degeneration over time with absence of responses by P490.	Slow degeneration with limited changes to retinal structure and function with both rod and cones similarly affected. Long-term assessment of optogenetic therapy applied at an early stage of disease would require extensive aging to determine efficacy over time.	Hawes et al. (2000), Kameya et al. (2002)	Not to date.
*Rd7*	*Nr2e3* Deletion of exons 4 and 5	Rosette formation of the outer nuclear layer begins by P13 but these resolve over time and are not present at 16 months of age. Outer nuclear layer and outer segments show reduced thickness compared to controls.	Rod and cone responses are normal at P30 and 5 months of age. Responses reduce to ∼50% compared to controls at 16 months of age.	Slow progressive photoreceptor degeneration with unusual early structural phenotype and aged mice showing only mild retinal degeneration.	Akhmedov et al. (2000)	Not to date.
*Rd8*	*Crb1* c.3481delC	Shortened inner and outer segments by P14. Subretinal spots appear that represent retinal disorganization and dysplasia apparent at P35.	ERG responses are attenuated compared to controls but not significantly so and are stable up to 12 months of age at which point progressive loss in function occurs.	Unusual structural changes, including to the inner layer, not reflective of a traditional retinitis pigmentosa phenotype and with limited functional defects.	Chang et al. (2002), Mehalow et al. (2003), Aleman et al. (2011)	Not to date.
*Rd9*	*Rpgr* 32bp duplication in ORF15	Normal structure up to 8 weeks of age with detectable differences in outer retinal thickness up to 12 months of age.	Normal rod and cone responses at 1 month of age with gradual age-related decline, responses detectable at 24 months of age.	Slow degeneration with limited changes to retinal function. The rate of degeneration is likely too slow to be appropriate for optogenetic strategies and is not characteristic of human late stage retinal degeneration.	Thompson et al. (2012)	Not to date.
*Rd10*	*Pde6b* R560C	Rod photoreceptor degeneration begins at P18 with complete loss by P35 with subsequent cone loss. By P45 only a single layer of cone cells remain.	Reduced but measurable scotopic ERG and good photopic ERG at P14-P28 with complete loss by P60.	With an early onset but mild rate of degeneration this model is more reflective of human disease and useful for long-term assessment of safety and efficacy.	Chang et al. (2002), Gargini et al. (2007)	Doroudchi et al. (2011), van Wyk et al. (2017)
*Rd12*	*Rpe65* c.130C > T	Normal appearance up to P40 with outer nuclear layer structure maintained to 3 months of age, at which point clear loss of outer segments occurs plus deposits in the RPE.	Rod ERG response significantly reduced by P21 and barely detectable at 8 months of age whilst cone responses are maintained.	Slow rate of structural changes yet early onset functional changes. This model offers the potential to target residual cones in a retina reflecting earlier stages of disease. The causative gene is important in RPE function and causes LCA therefore offers a different model in which to test optogenetic therapy.	Pang et al. (2005)	Not to date.
*Rd16*	*Cep290* Deletion of exons 35–39	Reduced outer nuclear layer thickness at P19 with only a single layer of cone cells by P45.	Reduced rod and cone functions at P18 with absent signals by P30.	Early onset with a rate of degeneration that falls between *rd1* and *rd10*, this offers the benefits of a good window of opportunity for intervention plus reduced aging requirements for assessments of efficacy.	Chang et al. (2006)	Doroudchi et al. (2011)
(*Nyx*^*nob*^)	*Nyx* 85bp deletion	Model of congenital stationary night blindness with a non-degenerate retina.	Normal a-wave but absent b-wave.	Of limited use for optogenetic studies due to abnormal bipolar cell function.	Pardue et al. (1998), Gregg et al. (2003)	Scalabrino et al. (2015)
**Transgenic**						
*Cpfl1/Rho^–/–^*	Cone photoreceptor function loss 1/rhodopsin double-knockout	Normal outer nuclear layer thickness at 3 weeks with ONL degenerating to one row of cell bodies by 10 to 12 weeks.	Loss of scotopic and photopic responses by week 12.	Good model reflecting retinitis pigmentosa through loss of photoreceptor cells whilst maintaining the inner retina and providing a good window for intervention.	Santos-Ferreira et al. (2016)	Santos-Ferreira et al. (2016)
*Opn4^–/–^, Gnat1^–/–^, Cnga3^–/–^*	*Opn4, Gnat, Cnga3* triple-knockout (TKO)	Normal retinal structure is maintained but functional loss occurs.	Response to flash ERG is achieved, considered to be due to stimulated rod opsin.	Mice lack optomotor responses and pupillary constriction to light yet maintain photoreceptor cells. This is an unusual model that displays abnormal function but not degeneration.	Hattar et al. (2003), Allen et al. (2010)	Lu et al. (2020)
*Rho^–/–^*	*Rho* Knockout	Normal outer nuclear layer thickness but an absence of outer segments at P24. Thinning begins at P30 and by P90 only a single row of cone cells remain with no outer segments.	Reduced rod responses by P24. Cone ERG maintained to P47 after which degeneration occurs.	Retinal degeneration with cone cell survival and function allowing for a big window of intervention with optogenetic therapy.	Humphries et al. (1997), Toda et al. (1999), Hassall et al. (2020)	Not to date.
*Nrl^–/–^*	*Nrl* Knockout	Absence of rods from birth with abnormal outer nuclear layer structure at P35. Surviving cones display reduced outer segment thickness and irregular outer nuclear layer stacking.	Absence of rod ERG function by P35. Cone responses enhanced.	Unusual structural changes to the outer nuclear layer and despite cone cell survival, the phenotype is not characteristic of typical retinitis pigmentosa.	Mears et al. (2001), Daniele et al. (2005)	Not to date.
*P23H/+*	*Rho* Knock-in of human P23H	Reduced outer nuclear layer thickness due to loss of rods at P63 with further loss to only 2–4 rows of nuclei at P112. Cone nuclei counts equivalent to age-matched control mice.	Scotopic ERG strongly reduced at P41 and barely detectable by P170. Photopic responses normal up to P40, mildly reduced at P70 and severely reduced by P170.	Slow, progressive degeneration, reflective of human RHO P23H retinitis pigmentosa and useful for assessment of safety and efficacy of optogenetic therapy in early stage retinal degeneration.	Sakami et al. (2011)	Not to date.
*Dp71^–/–^*	Knockout of the Duchenne muscular dystrophy small protein Dp71	Blood-retinal barrier permeability.	Slightly reduced ERG a-wave compared to WTs.	Of limited use for optogenetic strategies but useful comparisons for vector transduction studies in the presence of altered barriers.	Dalloz et al. (2003)	Vacca et al. (2013)

**Large animal models**						

Canine	e.g., Briard dog with a naturally occurring 4bp deletion in Rpe65	Progressive loss of photoreceptors over many years.	Reduced ERG.	Useful as a naturally occurring larger model of retinal degeneration.	Narfström et al. (1989), Wrigstad et al. (1992)	Ameline et al. (2017)
Non-human primates	Marmoset, Macaque	Normal.	Normal.	Useful for *in vivo* assessment of safety of optogenetic strategies with post-mortem evaluation of efficacy in the presence of pharmacological blockade of native photoreception.	Picaud et al. (2019)	Ivanova et al. (2010), Dalkara et al. (2013), Sengupta et al. (2016), Chaffiol et al. (2017), McGregor et al. (2020)

**Human-based organoids and explants**						

Human-derived organoids	Can be derived from iPSCs of normal controls or patients with inherited retinal disease.	Absence of outer segments when derived from normal controls.	Able to generate light-sensitive responses.	Being human-derived is advantageous to confirm efficacy of vectors in human cells but these take time to develop and convert from iPSCs (up to 200 days) and to then achieve evidence of transduction (up to 50 days), but functional outputs are possible.	Zhong et al. (2014), Reichman et al. (2017), Chichagova et al. (2019)	Gonzalez-Cordero et al. (2017), Khabou et al. (2018), Garita-Hernandez et al. (2020), Lane et al. (2020)
Human retinal explants		May be damage or scarred depending on the donor origin.		Restricted to donor availability, which may be of limited sample quality. Difficult to achieve any functional or quantitative output.	Orlans et al. (2018)	De Silva et al. (2016), van Wyk et al. (2017), Khabou et al. (2018)

*The asterisk indicates a premature stop codon/termination of the protein occurs. Broadly, the assessments include optimizations of vector and transgene biology and their transduction efficiency in target retinal cells, functional assessments of optogenetic molecules, as well as general safety of optogenetic gene therapy. These considerations will in turn determine the choice of a study model, or indeed assessments across multiple models within or across species.*

## Commonly Used Murine Models of Inherited Retinal Degeneration

The most historic model of inherited retinal disease is the naturally occurring *rd1* (Farber et al., 1994). Resulting from a mutation in the photoreceptor-specific gene *Pde6b*, this model is well characterized and displays a fast rate of degeneration in which most rod photoreceptor cells are lost by post-natal day 18 while the inner neural retinal structure is maintained with subsequent functional and morphological changes occurring over time (Strettoi et al., 2002). This rate of change is not equivalent to human disease, yet it is the most commonly used model for pre-clinical testing of optogenetic therapies. This is largely due to the fact that in a short time frame the mouse displays a retina lacking photoreceptor cells and that structurally reflects the later stages of human disease. The drawback of this rapid loss of photoreceptor cells is that it makes the *rd1* model less appropriate for testing optogenetic strategies aiming to rescue function from residual cone photoreceptor cells. Whereas cone photoreceptor cells can survive for many years following the loss of rod photoreceptor cells in human presentations of inherited retinal disease (Milam et al., 1998; Banin et al., 1999) and are therefore a potential target of optogenetic therapy, testing such an approach in *rd1* mice may be of limited use as they maintain only a single row of cone cells at 3 weeks of age. Although despite this, AAV targeting of residual cone photoreceptors with cone-specific promoters has been achieved with reporter EYFP expression observed in *rd1* mice up to 8 months of age (Busskamp et al., 2010). While residual cone photoreceptors were observed to survive in the *rd1* model, the opportunity for effective optogenetic intervention of cone photoreceptors may be greater in a slower model of degeneration. The health and function of cones in *rd1* mice may be compromised due to the early absence of rods, slower models of retinal degeneration should suffer less from these issues and therefore better reflect the degenerative status of the neural retina in human patients.

The *rd10* mouse offers an alternative slower model of retinal degeneration but also results from a mutation in *Pde6b*, although in this case it is a missense mutation that enables partial activity of Pde6b (Gargini et al., 2007). Though less commonly used than the *rd1* model, optogenetic strategies have been tested in *rd10* mice (Doroudchi et al., 2011; van Wyk et al., 2017). Whilst this model presents an early onset of retinal changes, the severity of disease is reduced compared to *rd1* and the rate of progression slower, providing greater opportunity for optogenetic intervention and long-term assessment. Other models of retinal degeneration offer similar opportunities, such as the naturally occurring *rd12*, *rd16* and the transgenic models *Rho^–/–^*, *Rho^*P23H/*+^*, and *Cpfl1/Rho^–/–^*. Consideration of the structural and functional features of retinal degeneration and the equivalent stage of human disease are important when assessing optogenetic therapy success. Another factor for consideration in pre-clinical testing in models of retinal degeneration is that one argument for use of optogenetic therapy is that the same vector may be applied to patients with various genetic origins of disease. To this end, initial testing may be best achieved in the *rd1* model with subsequent assessments performed in slower models of disease. Presentation of efficacy across different models of retinal disease would provide the strongest evidence of optogenetic success. Table 1 includes various models used in optogenetic studies to date and other potential models that could be explored in future studies.

## Large Animal Models

**FIGURE 2:**
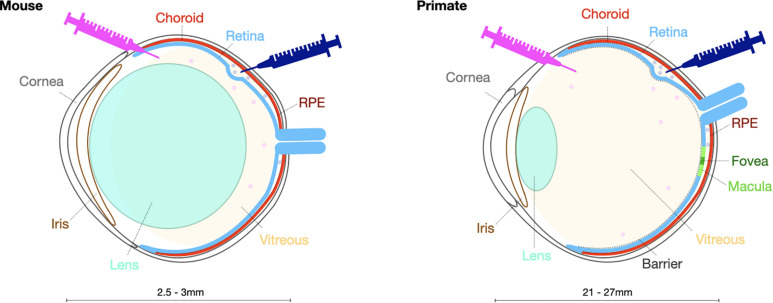
Schematic presentation highlighting key structural differences in the mouse and primate eye to be taken into account when selecting the surgical delivery method.

While murine models are commonly used in pre-clinical testing, large animal models such as pigs, dogs and non-human primates, have become increasingly necessary to investigate the safety and efficacy of potential optogenetic therapies prior to use in human trials. There are several important differences in ocular anatomy and physiology that make larger animal models more advantageous (Figure 2). First, the ocular size in a larger model enables investigations of intraocular vector delivery and development of surgical delivery techniques that would not be possible in the much smaller murine eye. In addition, the dimensions of the canine and non-human primate eyes enable intravitreal vector delivery that would have a comparable dilution effect to the pediatric human eye. Importantly, larger animals and specifically the primate retina has a thicker inner limiting membrane, a physical barrier separating the vitreous from the neural retina. In addition, canine and primate immune responses are likely to differ from murine models. These critical inter-species differences need to be taken into consideration when developing optogenetic vectors with ability to penetrate retinal cells from the vitreous.

In contrast to non-human primates, there are naturally occurring canine models of retinal degeneration, where progressive retinal atrophy secondary to mutations in, for example, *RPE65*, *XLPRA1* or *PDE6*β genes, displays great phenotypic similarities with equivalent retinal degeneration in humans (Beltran, 2009). On the other hand, the macula is only present in primates and has different cellular composition and anatomical arrangement, especially at the cone rich fovea where retinal ganglion cells and bipolar cells are displaced laterally. Canines nevertheless possess “area centralis” a central region of the retina with an increased density of cone photoreceptors. These important differences need to be considered when developing treatments for retinal degenerations that involve the macula, both in terms of cellular transduction profiles and functional assessments of ectopically expressed optogenetic molecules.

Several pre-clinical optogenetic studies have used canine and non-human primate models to assess the safety and expression profile of optogenetic vectors (Ivanova et al., 2010; Sengupta et al., 2016; Ameline et al., 2017) as well as the function and characteristics of optogenetic tools in terms of their ability to restore vision (Chaffiol et al., 2017; McGregor et al., 2020). Compared to murine models, limited *in vivo* (Ivanova et al., 2010; Chaffiol et al., 2017; McGregor et al., 2020) and *ex-vivo* (Sengupta et al., 2016) transduction of retinal ganglion cells was demonstrated in marmoset and macaque retinae, respectively, following delivery of AAV encoding microbial opsin-based sensors, with intra-ocular inflammatory responses observed in treated eyes (Chaffiol et al., 2017). In addition, despite long-term expression, similar findings of limited expression were observed in a canine model following treatment with a microbial and a human opsin (Ameline et al., 2017). Even the “state-of-the art” AAV vectors evolved for improved transduction in murine models (as will be discussed later in the review), demonstrate expression that is limited to a central para-foveal ring of retinal ganglion cells in a macaque retina following intraocular application (Dalkara et al., 2013). These results thus highlight the importance of testing optogenetic systems in primate models with outcomes most likely to mimic those of human subjects. However, these primate retinae are wild-type healthy retinae and not affected by retinal degeneration and remodeling that are likely to influence type of cell transduced and level of transgene expression within the cell. Additionally, healthy non-human primate retinae with a full set of photoreceptors make any functional electrophysiological or behavioral assessments of optogenetic therapies very challenging, although *in vitro* electrophysiology evaluation is achievable following pharmacological blockade of native photoreception (Chaffiol et al., 2017).

## Human-Derived Retinal Organoids

Use of model organisms serve several purposes and the benefits of *in vivo* testing are many, particularly in the field of optogenetics where assessment of visual function and processing can be achieved. However, it is important to consider the benefits of other models in pre-clinical testing. In recent years, protocols for generating retinal organoids from human iPSCs have been developed, which enable formation of cultured structures derived from human cells that reflect the cell types and organization of a neural retina (Zhong et al., 2014; Reichman et al., 2017; Quinn et al., 2018). These retinal organoids provide an incredibly useful tool not only for optogenetic therapy pre-clinical testing but also in general for the field of retinal gene therapy. As described above, not all animal models replicate effectively human disease states therefore testing of gene therapy vectors can be challenging. For example, the *Rp2^–/–^* mouse model poorly replicates human retinal disease caused by absence of RP2 function. Generation of retinal organoids from patient-derived samples was recently described and these proved a useful *in vitro* model for assessing *RP2* gene therapy rescue (Lane et al., 2020). For optogenetic strategies, retinal organoids also provide an interesting model for pre-clinical testing. Whilst they do not perfectly replicate the neural retina, distinctive layers of different cell types have been shown across different research groups using similar protocols (Zhong et al., 2014; Reichman et al., 2017; Quinn et al., 2018; Chichagova et al., 2019). A consistent feature across all protocols appears to be a lack of photoreceptor outer segment structures, but this does not pose a problem for testing of optogenetic vectors as indeed it mimics the absence of such structures in retinitis pigmentosa.

The initial challenge of testing any gene therapy strategy is to achieve transduction of the desired cell types. Vector tropisms and photoreceptor-specific promoters have been tested in retinal organoids (Gonzalez-Cordero et al., 2017; Garita-Hernandez et al., 2020). Current data suggest a large number of AAV capsid serotypes offer relatively poor transduction efficiencies (2–20%), including AAV2, ShH10, AAV5, AAV8, AAV8 Y733F, and AAV9 with the most encouraging variant appearing to be AAV2(7m8), achieving up to 60% transduction efficiency (Gonzalez-Cordero et al., 2017; Khabou et al., 2018; Garita-Hernandez et al., 2020). Differentiation of retinal organoids is a long process and can take up to 140 days with vector application possible during early stages of the differentiation process (Garita-Hernandez et al., 2020) or at the later stages of differentiation (Lane et al., 2020). The extensive protocols and long experimental time frames required for retinal organoid differentiation and the subsequent transduction reflect the inhibitory aspects of using these for pre-clinical testing, but the benefits of testing in human-derived samples may outweigh such issues.

A successful optogenetic strategy will depend on achieving opsin localization in the membrane of the targeted cell type whilst avoiding build-up inside the cell. It has been indicated that while delivery and expression of a variety of different microbial opsins can be achieved in retinal organoids, not all localized well to the membrane with endoplasmic reticulum accumulation and unfolded protein responses mechanisms observed (Garita-Hernandez et al., 2020). For the opsin variants that trafficked well to the membrane of targeted photoreceptor cells, functional activation was achieved. In addition, any mutagenic changes within protein structures aimed to improve functional properties of optogenetic molecules (e.g., making them more light sensitive) may have deleterious effect on protein trafficking and expression within cell membrane. In general, human cone opsin mutations can lead to accumulation within the cells and an absence of functional response (McClements et al., 2013) and rhodopsin mutations may impair folding and trafficking and lead to cell death (Athanasiou et al., 2017). While pre-clinical testing of optogenetic strategies in retinal organoids is still a relatively new technique, the data achieved so far indicate it offers an exciting addition to the development and assessment of such therapies.

## Human Retinal Explants

A final model for consideration when conducting pre-clinical testing involves the use of human retinal explants. These can be obtained and cultured post-mortem (van Wyk et al., 2017; Khabou et al., 2018) or following an emergency retinectomy (De Silva et al., 2016) and may be derived from human or non-human primates (Hickey et al., 2017). Whilst useful as a screening tool to observe successful transduction of human cells, the health of such explants can be problematic and maintaining survival in culture long enough to observe successful transduction can be an issue. Additionally, the cell types that can be transduced in human retinal explants appear to be limited with no detectable expression observed in ON-bipolar cells, most likely due to downregulation of bipolar cell specific gene expression in the explant system (van Wyk et al., 2017). With human retinal explants unlikely to provide any quantitative or functional outputs following gene therapy treatment, retinal organoids may provide a more appropriate model for *in vitro* testing of optogenetic vectors.

## Cell-Specific Targeting in the Degenerate Retina

### Vector Considerations

**FIGURE 3:**
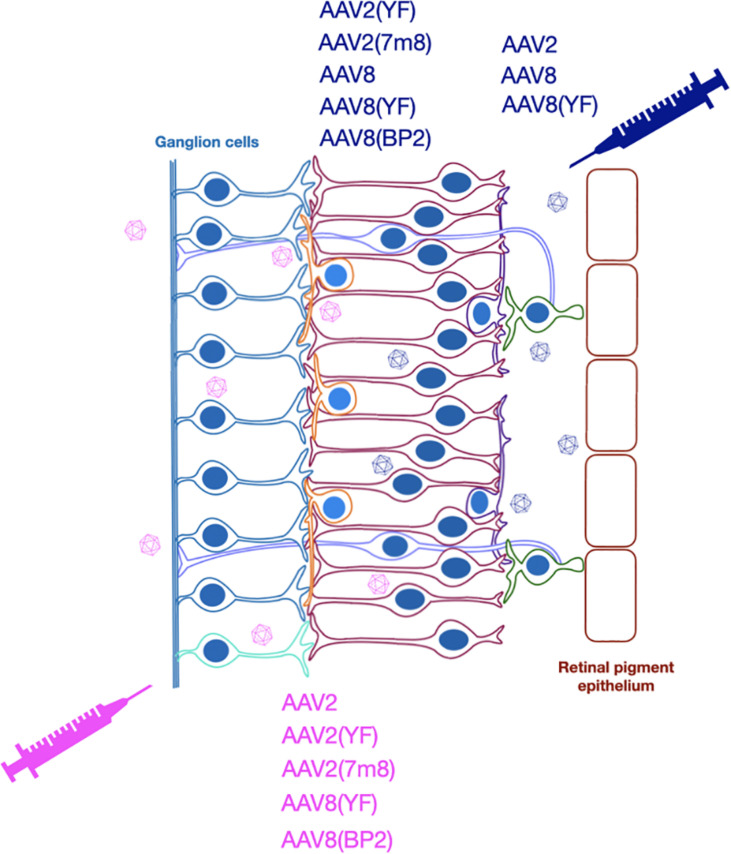
Optogenetic strategies have used subretinal or intravitreal injection to deliver various AAV vectors for expression of different opsins.

While optogenetic strategies are not intended to be restricted by the original genetic cause of degeneration, the stage of disease will influence the type of suitable vector and hence key features of vector design. To be successful, optogenetic therapy requires expression of a light-sensitive opsin molecule in the membranes of the surviving cells of the retina and achieving this relies on efficient delivery and expression of an optogenetic transgene to target cells. AAV vectors have been the primary vector of choice over the past decade and several AAV vectors have now been used in numerous clinical trials for a wide variety of retinal diseases (Bainbridge et al., 2015; Russell et al., 2017; Xue et al., 2018; Cehajic-Kapetanovic et al., 2020b), the majority being single gene diseases. Indeed, the first approved human AAV gene therapy (Luxturna) is for the treatment of an inherited retinal disease, Leber congenital amaurosis. While systemic delivery of AAV vectors has proved more challenging and recently resulted in the tragic deaths of trial participants (Wilson and Flotte, 2020), their use in the eye has been shown to cause minimal adverse events in clinical trials (Nuzbrokh et al., 2020). While lentiviral vectors can struggle to achieve good transduction efficiency and with transgene expression typically only observed around the site of injection (Balaggan and Ali, 2012; Puppo et al., 2014), AAV delivery can achieve transduction patterns exceeding the area of the bleb (Boye et al., 2016; Yiu et al., 2020). AAV vectors also offer flexibility on the delivery route used, namely intravitreal or subretinal (Figures 2, 3).

Currently, the main limitations of AAV vector use for treating inherited retinal disease appear to be their packaging capacity and ability to favor transduction of particular cell types. For optogenetic transgenes, the packaging capacity is not likely to be an issue as opsin coding sequences are relatively small compared to genes used in gene supplementation strategies, such as *RPGR* (Fischer et al., 2017). However, AAV vectors for optogenetic strategies have benefited in recent years from development of engineered capsid variants. While unnecessary for current clinical trials targeting photoreceptors or cells of the retinal pigment epithelium, for which AAV8 and AAV2 variants have proven effective (Xue et al., 2018; Cehajic-Kapetanovic et al., 2020b), the requirement of optogenetic therapies to target residual cone photoreceptors, inner retinal layers and the outer ganglion cell layer has required investigation of new capsid variants. As previously discussed, cone photoreceptor cells can survive for many years after the loss of the rod photoreceptor cells and despite the loss of outer segments, and therefore light sensitivity (Milam et al., 1998; Banin et al., 1999), they are nevertheless considered an optogenetic target. Historically, efficient transduction in cones has proven difficult relative to rod photoreceptors (Mussolino et al., 2011; Boye et al., 2012) but recent studies with new capsid variants have increased potency (Khabou et al., 2018). Successful reporter transgene expression was shown in the cone photoreceptors of *rd10* mice as well as retinal organoids, human post-mortem explants and in non-human primates (Khabou et al., 2018). However, despite the improvements in cone photoreceptor targeting abilities for pre-clinical testing, these cell types will not be appropriate targets for all cases of late stage retinitis pigmentosa. Although the cones may survive, targeting a cell of compromised health and structure may not provide the best long-term option and indeed in some patients, they may not maintain enough surviving cones for optogenetic therapy of these cells to be worthwhile. Delivery of optogenetic transgenes to residual cone photoreceptors in *rd1* mice enabled restoration of visual function (Busskamp et al., 2010) and while this is encouraging, it should be considered that the pattern of cone density in mice is different to humans. In humans, cone photoreceptors are enriched in the macula region and reduce in density outwardly from this point, therefore targeting such cells will make use only of a small central region of the retina.

Optogenetic strategies that target the inner layer of the retina or ganglion cell layer have potential to enable a broader area of induced photosensitivity. Achieving efficient transduction of the inner retinal layers has largely relied on engineered and novel AAV capsid serotypes as opposed to wild type forms that have struggled to transduce the inner retina (Charbel Issa et al., 2013). Increased tropism and transduction success of the retinal layers has been achieved by mutation of multiple surface tyrosine (Y) residues to phenylalanine (F). While single residue Y to F changes do not effectively enable inner retinal targeting (De Silva et al., 2016), multiple residue mutations in AAV2(2–4YF) have shown more success in both in wild type mice (Petrs-Silva et al., 2011) and mice with retinal degeneration (Gaub et al., 2015). Furthermore, a directed evolution approach has created the AAV2(7m8) variant featuring a seven amino acid insertion in capsid protein VP1 (Dalkara et al., 2013). The AAV2(4YF) and AAV2(7m8) variants provided similar transduction profiles when directly compared following subretinal and intravitreal injection in the *rd1* mouse and included transduction of the inner retina (Hickey et al., 2017). However, expression in bipolar cells was not robust, indicating that while successful, further improvements in transduction may be required.

A particularly promising AAV capsid option for optogenetic approaches targeting the inner retina is the AAV8(BP2) variant. This was created by directed selection of a library of AAV8 capsid mutants focused on a variable region of nine amino acids, considered important for receptor attachment and transduction (Cronin et al., 2014). This AAV8(BP2) capsid provided good transduction of ON-bipolar cells, including limited expression in non-diseased human retinal explants with a strong pan-neuronal CMV promoter. This was the first demonstration of robust bipolar cell transduction from an AAV vector but transduction in a wild type mouse does not necessarily translate to degenerate retina or indeed non-human primate and human retina. Further confirmation of human bipolar cell transduction was provided in a later study using human retinal explants that also showed targeting of cone ON-bipolar cells by a reporter AAV8(BP2) vector (van Wyk et al., 2017). This study showed comparable human bipolar cell expression patterns were achieved from both AAV8(BP2) and AAV2(7m8) vectors and also performed similarly in the degenerate 11-week-old *rd10* mice. Such developments in capsids have advanced the potential of optogenetic strategies by enabling transduction in bipolar cells.

Transduction of the innermost layer of the retina, the retinal ganglion cells has been readily achieved in mouse models following intravitreal injection (Yin et al., 2011; Smith and Chauhan, 2018). Wild type AAV capsid transduction appears limited to the retinal ganglion cells unless applying an additional adjunctive (Cehajic-Kapetanovic et al., 2011, 2018) whereas modified AAV capsids penetrate further following intravitreal injection into the inner retina (Kay et al., 2013; Hickey et al., 2017; van Wyk et al., 2017).

### Transgene Considerations

While the first hurdle in any gene therapy strategy is getting the AAV to enter the desired cell type, transgene design can influence the success of subsequent expression. In addition to being difficult to target with AAV, cone photoreceptor cells have also proved difficult in the development of efficient cell-specific promoters (Li et al., 2008) but the development of a 1.7kb version of the human red/green cone opsin promoter, known as PR1.7, was shown to achieve robust and selective expression in cone photoreceptor cells (Ye et al., 2016; Khabou et al., 2018). Utilizing the AAV2(7m8) capsid and an intravitreal injection route, the latter research group found the PR1.7 promoter to be more specific than the mouse cone arrestin (mCAR) promoter, which resulted in expression in both rod and cone photoreceptor cells. Cell-specific promoters are often considered to pose a risk in gene therapy strategies in case of changes in gene expression profiles in degenerating cells. This may be particularly concerning for optogenetic strategies targeting residual cone photoreceptor cells, which have a changing profile of expression, function and morphology over time (Hassall et al., 2020). Despite these concerns, successful expression from the PR1.7 promoter was observed 2 months post-injection in *rd10* mice though how this might translate to humans should be approached with caution.

Following loss of photoreceptors in the degenerating retina, the secondary neurons of the retina, the horizontal and the bipolar cells are considered as the next best option for optogenetic approaches. Transducing the distal retinal circuitry has greater chance of preserving upstream retinal processing, and the ON-bipolar cell-specific expression is thought to be particularly important in achieving this. In a normal retina, the bipolar cells transfer the light signals received from photoreceptor cells to amacrine and retinal ganglion cells (Figure 1). Morphological comparisons have determined there to be just one rod-bipolar cell type but multiple cone-bipolar cell types (Wässle et al., 2009) and these fall into two broad functional categories of ON (rod and cone) and OFF (cone only) (Nelson and Connaughton, 2007). The ON-bipolar cells are depolarized by light-stimulated photoreceptor cells whilst OFF-bipolar cells are hyperpolarized in the same receptive field. Selective expression of opsins in ON-bipolar cells is considered important to avoid interfering with the complex interconnected signaling pathways of the remaining neural retina. Making both ON- and OFF-bipolar cells respond to light in the same way could result in ambiguous signals, although cortical plasticity could play a role in filtering out the noise, as seen with subretinal implants (Cehajic-Kapetanovic et al., 2020a). A combination of effective AAV transduction and promoter for selective expression of opsins in ON-bipolar cells are therefore highly desirable features of optogenetic therapies.

To date, *4xGrm6-SV40* is the most studied promoter for optogenetic strategies aiming to achieve opsin expression in ON-bipolar cells. The metabotropic glutamate receptor type 6 (mGluR6) receptor was found to be associated with ON-bipolar cell activity (Nakajima et al., 1993; Nomura et al., 1994). A critical 200bp enhancer region in the mouse *Grm6* gene that encodes mGluR6 was subsequently paired with the SV40 promoter to achieve ON-bipolar cell specific expression of a reporter gene (Kim et al., 2008). This has since been used with success by other research groups in their optogenetic approaches (Lagali et al., 2008; Doroudchi et al., 2011; Cehajic-Kapetanovic et al., 2015; Macé et al., 2015). A comparison of multiple copies of the *Grm6* enhancer identified that including four copies prior to the SV40 promoter improved reporter gene expression (Cronin et al., 2014). Whilst this promoter has been shown to primarily enable transgene expression in ON-bipolar cells, expression has been identified in other cell types (van Wyk et al., 2015, 2017). The expression profile of an optogenetic transgene with fluorescent reporter revealed predominant expression in amacrine cells and a lack of ON-bipolar cell targeting in *rd1* mice. In wild type mice, amacrine cells were also the most targeted cell type when using the AAV2(7m8) yet when the AAV8(BP2) capsid was used, multiple cell types revealed reporter transgene expression, including ON-bipolar cells. Interestingly, a similar expression profile was then observed in *rd10* mice yet not in *rd1* mice, even when injected at earlier age. This highlights the consideration raised earlier regarding the need to use multiple models of retinal degeneration when assessing a given optogenetic therapy. Furthermore, the difference in expression profile from a given promoter varies between mouse models and is to an extent dependent on other vector selections, such as the capsid.

Use of an extended *mGluR6* promoter containing additional intronic sequence enabled enhanced reporter gene expression in ON-bipolar cells in mice and non-human primates (Lu et al., 2016). However, this promoter variant is considerably longer than the commonly used variant and therefore potentially less desirable as this can be limiting to transgene design. Transgenes for AAV vectors cannot exceed the packaging capacity of AAV, which is typically ∼4.8 kb. The majority of opsin coding sequences are relatively small (∼2 kb) enabling room for a larger promoter. However, other transgene elements may be desirable to enhance expression, such as inclusion of a WPRE (Patrício et al., 2017) or fluorescent marker to create a fusion protein. Additional membrane trafficking signals may also be required (discussed later within opsin considerations). Therefore, the larger *mGluR6* promoter may not prove to be a limiting factor in its future use in optogenetic transgenes but it will be dependent on other factors of transgene design that may prove critical for a given optogenetic strategy.

A mini-promoter named Ple155 (derived from the *PCP2* gene) has also been shown to provide ON-bipolar cell expression in wild-type mice (De Leeuw et al., 2014) and was used to restore vision in a mouse model (*Nyx^–/–^*) of congenital stationary night blindness and non-degenerated retina (Scalabrino et al., 2015). Whilst the *Nyx^–/–^* mouse model is not of relevance to optogenetic strategies, *NYX* is a bipolar cell-specific gene and is therefore of interest in its use as an alternative ON-bipolar specific promoter. Interestingly, ON-bipolar cell specific expression was achieved only when using the Ple155 promoter in combination with an AAV2 (and not AAV8) vector and only in very young mice (at P2 and not at P30) with undifferentiated bipolar cells. It therefore remains to be seen whether the Ple155 promoter offers any advantage in ON-bipolar cell targeting over the *4xGrm6-SV40* in degenerate retina and whether ON-bipolar cell transduction can be achieved with either promoter in non-human primate or indeed in human retina.

Targeting of bipolar cells may be preferable in the earlier stages of retinal degeneration but in later stages of disease, these cells can become disordered and also degenerate (Pfeiffer et al., 2020). These changes include alterations in the neural connections made between cell types of the neural retina structure but also their morphology and general structural layout, Figure 1. Not all patients will present in clinics at the earlier stages of disease, therefore some optogenetic approaches aim to target the retinal ganglion cells, which remain most resistant to degeneration retaining their physiological properties and laminar location in the retina. In mouse models, this is achieved relatively easily with multiple AAV serotypes by intravitreal injection and gene expression can be restricted using the synapsin-1 promoter (*Syn1*) (Sengupta et al., 2016) or the neurofilament heavy polypeptide promoter (*Nefh*) (Hanlon et al., 2017). Given the preference for transduction of ganglion cells when using the intravitreal injection route, ubiquitous promoters have also been used with success (Bi et al., 2006; Bin Lin et al., 2008; Zhang et al., 2009; Liu et al., 2016). However, the risks of introducing light-sensitive responses to more than one cell type are currently unknown and may interfere with the interconnectivity of the complex neural circuits of the retina.

## Opsins for Optogenetic Therapy

**FIGURE 4:**
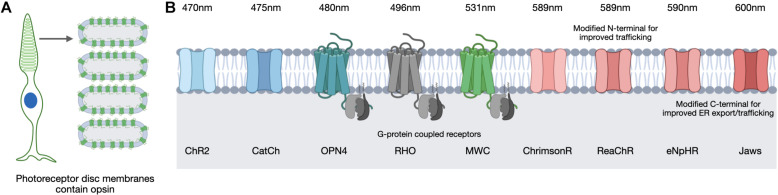
In the human retina, light-sensitive opsins for vision are located in the photoreceptor cells on the membranes of specialized discs **(A)**. Optogenetic strategies use multiple opsin variants of microbial and vertebrate origin that are sensitive to different peak wavelengths **(B)**. Images created using BioRender.com.

The opsins used for optogenetic approaches include both microbial and vertebrate varieties (Figure 4). These have different characteristics and vary in many ways including their sensitivity to light, recovery rates and in the type of response they elicit (Simon et al., 2020). Opsins are transmembrane proteins that absorb light and in the case of vertebrate opsins, they are G-protein coupled receptors whose activation leads to cGMP-gated cation channels closing and a subsequent hyperpolarization of the photoreceptor cell (Figure 4). In contrast, microbial opsins are light-activated ion channels rather than G-protein coupled receptors that can cause depolarization or hyperpolarization depending on the nature of the ion channel. It is for this reason that the microbial opsins have proven popular for optogenetic strategies as they can directly influence the cell polarization in response to light without the need for other G-protein cascade elements. This may prove important in successfully converting cells that were not intended to be light sensitive.

Opsin selection requires consideration of various factors related to opsin function, including the conductance rate and ion selectivity. For example, microbial opsins exhibit differences in their conductance of cations or anions and in the rate of ion influx achieved upon light activation. Considerations of opsin kinetics are also important as the timing of a channel opening and closing to enable ion influx will influence the extent of a response. Opsins differ in the intensity of the light required to induce a response and the peak wavelength of light they respond to (Figure 4). The intensity of light necessary to induce stimulation is of particular safety importance as the retina can be damaged by certain light intensities and wavelengths (Youssef et al., 2011). Natural light provides a general exposure range of 10^–4^ to 10^5^ lux and safety thresholds for humans have been defined (The European Parliament and the Council of the European Union, 2006; ICNIRP, 2013; van Norren and Vos, 2015) therefore an optogenetic therapy would need to be successful within these limits. The position of the opsin is also critical in order to be optimally exposed to the light source and in a great enough proportion to achieve an appropriate response without inducing membrane instability or overexpression. Finally, opsins can suffer desensitization, in which they become less responsive to light after repeated stimulation and therefore require recovery time prior to their next light-activation. This is also referred to as bleaching and opsins vary in the extent to which they suffer and recover from this process, for example, rhodopsin of the rod photoreceptors is particularly susceptible and suffers reduced phototransduction capacity following exposure to bright light (Pepperberg, 2003). These features are all important considerations and will be referred to throughout the following discussion of opsin candidates for optogenetic therapeutic approaches.

## Microbial Opsins

The first microbial opsin identified for use as an optogenetic tool was channelrhodopsin-2 (ChR2) (Boyden et al., 2005), which causes entry of cations into the cell resulting in depolarization. It has been the most commonly used microbial opsin to date and is induced by blue light (470 nm). It was used in the first pre-clinical optogenetic strategy for rescuing visual function to the *rd1* mouse (Bi et al., 2006) and a subsequent humanized version (containing an H143R mutation) fused to GFP has been stably expressed using the *Grm6-SV40* promoter in *rd1* mice following subretinal injection (Doroudchi et al., 2011) and intravitreal injection (Macé et al., 2015). In the latter approach, both ON and OFF visual responses were detected at 4–10 weeks post-injection, indicating its potential for optogenetic therapy.

An engineered variant of ChR2 carrying an L132C mutation was developed and named CatCh as it is a calcium translocating channelrhodopsin and has nearly six-fold enhanced Ca^2+^ permeability relative to ChR2, which improved its sensitivity to light and kinetics (Kleinlogel et al., 2011). An optogenetic transgene containing an EGFP fusion of this protein was expressed in ON-bipolar cells in *rd1* mice and incurred light-induced ganglion cell responses 4 weeks post-injection (Cronin et al., 2014). A red-light activated depolarizing ChR (ReaChR) was generated with a peak spectral sensitivity of 590nm and this ReaChR included an *N*-terminal sequence to aid membrane trafficking (Lin et al., 2013). When expressed specifically in retinal ganglion cells using the *SYN1* promoter following AAV2 intravitreal delivery in *rd1* mice, visual and behavioral responses to light were observed at light intensities within safety thresholds (Sengupta et al., 2016). An algal-derived variant similar to ReaChR is ChrimsonR (Klapoetke et al., 2014). Comparisons of ReaChR and ChrimsonR localization and function in human retinal organoids revealed better trafficking of ReaChR to the cell membrane than ChrimsonR but both provided detectable responses to light stimulation (Garita-Hernandez et al., 2018). Despite the reduced trafficking efficiency, ChrimsonR did not accumulate excessively in the cell organelles or co-localize with markers of endoplasmic reticulum retention or the unfolded protein response.

Other microbial opsins being used for optogenetic strategies are light-activated hyperpolarizing chloride pumps, of which halorhodopsin (NpHR) was the first to be reported and is sensitive to yellow light (Zhang et al., 2007). Hyperpolarizing opsins are of particular interest in optogenetic strategies targeting surviving cone photoreceptor cells, which as described above, can continue to exist in a dormant state in the degenerate retina despite loss of cone-mediated light responses (Lin et al., 2009; Busskamp et al., 2010). NpHR has a peak wavelength sensitivity of 589 nm and light activation leads to hyperpolarization of the cell, mimicking the native response of a photoreceptor cell. However, this protein was found to accumulate and form unwanted aggregates when expressed at high levels (Gradinaru et al., 2007) so an enhanced version, eNpHR, was created (Gradinaru et al., 2008). The addition of an *N*-terminal signal peptide and a *C*-terminal endoplasmic reticulum export signal improved the membrane localization of this hyperpolarizing opsin. AAV delivery of eNpHR using photoreceptor-specific promoters achieved electrophysiological responses to light in *rd1* mice equivalent to those in wild type mice (Busskamp et al., 2010). Further improvements to eNpHR were made by way of additional trafficking signals to provide variant eNpHR 3.0 (Gradinaru et al., 2010).

Despite the addition of these signals, testing *in vitro* did not reveal membrane localization, however, testing in human retinal organoids did achieve membrane localization and subsequent light responses (Garita-Hernandez et al., 2018). However, despite the addition of export and localization signals, these comparisons in human retinal organoids indicated the hyperpolarizing opsin Jaws showed better membrane localization relative to eNpHR 3.0. Jaws is an engineered chloride pump cruxhalorhodopsin that is red-shifted by 14 nm relative to the eNpHR variants and therefore has a peak sensitivity of 600 nm (Chuong et al., 2014). AAV delivery in *rd1* mice of a Jaws transgene driven by the mCAR promoter achieved expression 4 weeks post-injection and achieved spike responses from isolated ON- and OFF-retinal ganglion cells following light stimulation at 600 nm (Chuong et al., 2014). It was also observed that responses could be achieved from 550 nm (green) and 470 nm (blue) wavelengths and that Jaws provided more retinal ganglion cell spiking than eNpHR over a broader light spectrum, which suggest Jaws may be more suitable for use in human optogenetic therapy.

The eNpHR 2.0 variant has been used in combination with a human rhodopsin promoter to achieve expression in rod photoreceptors of wild type mice, which were then harvested and the eNpHR 2.0-expressing rod photoreceptor cells subsequently isolated and injected into *Cpfl1/Rho^–/–^* female mice (Garita-Hernandez et al., 2019). Responses to 580 nm light were achieved as were spike recordings of ON- and OFF-retinal ganglion cells, indicating connections were made between the transplanted cells and the surviving secondary neurons of the host retina. Encouragingly, light intensities within the safe limits defined for the human eye successfully stimulated responses and light avoidance behavior in treated mice was also observed. This same study also induced Jaws expression from the mCAR promoter in human iPSC-derived cone photoreceptor cells, which were then injected into *Cpfl1/Rho^–/–^* and *rd1* mice. Robust photocurrents were achieved, which peaked when stimulated with a wavelength of 575 nm. As for eNpHR stimulation, responses were achieved from light intensities below safety thresholds and both ON- and OFF-retinal ganglion cell responses were achieved. Jaws has also been shown to have good membrane localization in cone photoreceptor cells of non-human primates following delivery of an AAV9(7m8) vector with the PR1.7 promoter (Khabou et al., 2018). These studies highlight the potential for both eNpHR and, in particular, Jaws to be used in future human clinical trials for optogenetic stimulation of residual cone cells.

## Vertebrate Opsins

Despite the encouraging data from studies using microbial opsins use of a native human opsin may be more desirable as it poses less risk of immune reaction. Both human and mouse retinal cells produce five main opsin variants that respond to different wavelengths of light: rhodopsin, expressed by rod photoreceptor cells (RHO); short-wave cone opsin (SWC), medium-wave cone opsin (MWC), and long-wave cone opsin (LWC), expressed by cone photoreceptor cells; and finally, melanopsin (OPN4). This latter opsin is not essential for vision but instead is an important sensor for the circadian clock and is expressed by a sub-population of intrinsically photosensitive retinal ganglion cells (ipRGCs). One of the first optogenetic studies to use a native opsin for pre-clinical testing in *rd1* mice boosted the melanopsin expression with rescue of basic visual functions (Bin Lin et al., 2008). Subsequently, human melanopsin expression in cells of the inner retina of *rd1* mice has also been achieved (Bin Lin et al., 2008; Liu et al., 2016; De Silva et al., 2017). These studies reported greater light sensitivity compared to the microbial opsins with rescue of visual responses achieved up to 12 months post-injection (De Silva et al., 2017). A further study created a chimera of mouse melanopsin and the mGluR6 receptor, Opto-mGluR6, under the control of the *Grm6-SV40* promoter in order to test the melanopsin kinetics in ON-bipolar cells in a transgenic mouse model (van Wyk et al., 2015). Encouragingly, transduction of only a small fraction of ON-bipolar cells (∼12%) using AAV2(4YF) vector delivery, was sufficient to induce light responses in retinal ganglion cells.

Success in restoring responses to light in the degenerate retina with human opsins have also been achieved with ectopic expression of native rod opsin, rhodopsin. Intravitreal delivery of AAV2 achieved human rhodopsin expression in ON-bipolar cells of *rd1* mice and improved visual and behavioral responses 8–12 weeks post-injection including resolution of flicker, coarse spatial patterns and elements of natural scene (Cehajic-Kapetanovic et al., 2015; Eleftheriou et al., 2017). Encouraging data were also achieved with the same transgene in *rd1* mice but using an AAV2(4YF) vector (Gaub et al., 2015). The responses of rhodopsin-treated mice were compared to those treated with an identical vector delivering humanized ChR2, and rhodopsin was found to offer greater and more sensitive responses than ChR2. The same study group recently expressed vertebrate MWC in the retinal ganglion cells of *rd1* mice (Berry et al., 2019) with behavioral tests showing signs of functional rescue. The speed and sensitivity of response to light of MWC was improved compared to rhodopsin function in ganglion cells, but it remains to be seen how the function of cone photopigments compares to rhodopsin kinetics in ON-bipolar cells. Ultimately, human trials are needed to determine how these ectopically expressed human opsins compare to visual function achieved by native rod and cone opsins in photoreceptor cells.

## Discussion

The pre-clinical work to date demonstrates that optogenetic strategies are able to restore vision to the degenerate retina using a multitude of transgene and vector combinations. Development of new capsid varieties has greatly aided transduction success of the inner retina and improved cell-specific expression profiles. Progress has been made in understanding visual and behavioral responses following delivery of a wide variety of opsins in various cell types of the surviving retina in animal models. The major barrier to translation of many of these optogenetic therapies has been the lack of evidence of efficient optogene expression in healthy non-human primate, or indeed degenerate human retina, using the available tools.

None-the-less, both ChR2 and ChrimsonR are currently being tested in Phase I/IIa clinical trials. The NCT02556736 trial describes delivering ChR2 primarily to retinal ganglion cells using AAV2 intravitreal injection in patients with advanced retinitis pigmentosa. This trial was initiated by Retrosense Therapeutics (now part of Allergan) and is an open-label, dose escalation study that began recruiting in 2015 but to date, no data have been released. A more recent trial initiated by GenSight Biologics in 2017 (NCT03326336) also involves intravitreal delivery but of an AAV2(7m8) vector aiming to express the ChrimsonR-tdTomato fusion protein. This PIONEER trial by GenSight is not only of interest because it is the first to use the capsid variant AAV2(7m8) for expression of ChrimsonR-tdTomato, but also because it combines the gene therapy with a specialized wearable visual stimulation device. Given the considerations discussed earlier in this review regarding the microbial opsins, the selection of ChrimsonR instead of ChR2 offers a safer and potentially more sensitive opsin than the first trial using ChR2. The inclusion of tdTomato is intriguing as this is a fluorescent marker used to confirm transgene expression and provides no functional benefit. Typically, such markers are used for pre-clinical studies and are then removed prior to use in human clinical trials. In this particular study design, the confirmation of ChrimsonR expression by detection of tdTomato enables researchers to be confident successful transduction has occurred and that the visual stimulation device can subsequently be used. This is an interesting step forward in gene therapy transgene design for human clinical trials and it may be that such implementation of fluorescent reporters may be used more often in the future. However, caution should always be applied in expressing proteins in human cells if they are not necessarily required to achieve a therapeutic outcome. This year, press releases of preliminary clinical trial safety data have also been provided, suggesting good tolerance of the vector so far (gensight-biologics.com). However, it remains to be seen what the long-term effects of expressing an algal-derived protein on the membranes of retinal cells are and whether these may be detected by the immune system and therefore lead to an undesired immune response against the transgene product. It is clear though that the current dose-escalation study is a vital step forward in providing such treatments to individuals with severe retinal degeneration and limited light perception.

Given the limited light sensitivity of current microbial opsins, the need for an additional device and the potential for immune responses triggered by foreign proteins, it could be that future therapies with human opsins provide a better opportunity for success. It is worth noting that Acucela Inc (now Kubota Vision) are currently developing human rhodopsin optogenetic therapy as are Applied Genetic Technologies Corporation (AGTC) in collaboration with Bionic Sight but the details of the latter treatment are currently unavailable.

In summary, studies to date indicate optogenetic therapies show great therapeutic potential for restoring vision in patients with advanced inherited retinal disease and more human trials are necessary as the next major step in advancing the field. It remains to be seen how the human visual system, affected by degeneration and remodeling, responds to optogenetic stimulation at the level of retinal photoreception and by means of cortical plasticity for improved and meaningful visual perception.

## Author Contributions

MM drafted, edited, and created figures for the manuscript. FS reviewed and edited the manuscript. RM provided funding and critical review of the manuscript. JC-K initiated the manuscript and provided critical review. All authors contributed to the article and approved the submitted version.

## Conflict of Interest

The authors declare that the research was conducted in the absence of any commercial or financial relationships that could be construed as a potential conflict of interest.
